# Selective elimination of neuroblastoma cells by synergistic effect of Akt kinase inhibitor and tetrathiomolybdate

**DOI:** 10.1111/jcmm.13106

**Published:** 2017-02-28

**Authors:** Jarmila Navrátilová, Martina Karasová, Martina Kohutková Lánová, Ludmila Jiráková, Zuzana Budková, Jiří Pacherník, Jan Šmarda, Petr Beneš

**Affiliations:** ^1^ Department of Experimental Biology Faculty of Science Masaryk University Brno Czech Republic; ^2^ Center for Biological and Cellular Engineering International Clinical Research Center St. Anne's University Hospital Brno Czech Republic

**Keywords:** neuroblastoma, tetrathiomolybdate, Akt kinase, cell viability, glycolysis, oxygen consumption, metabolic plasticity

## Abstract

Neuroblastoma is the most common extracranial solid tumour of infancy. Pathological activation of glucose consumption, glycolysis and glycolysis‐activating Akt kinase occur frequently in neuroblastoma cells, and these changes correlate with poor prognosis of patients. Therefore, several inhibitors of glucose utilization and the Akt kinase activity are in preclinical trials as potential anti‐cancer drugs. However, metabolic plasticity of cancer cells might undermine efficacy of this approach. In this work, we identified oxidative phosphorylation as compensatory mechanism preserving viability of neuroblastoma cells with inhibited glucose uptake/Akt kinase. It was oxidative phosphorylation that maintained intracellular level of ATP and proliferative capacity of these cells. The oxidative phosphorylation inhibitors (rotenone, tetrathiomolybdate) synergized with inhibitor of the Akt kinase/glucose uptake in down‐regulation of both viability of neuroblastoma cells and clonogenic potential of cells forming neuroblastoma spheroids. Interestingly, tetrathiomolybdate acted as highly specific inhibitor of oxygen consumption and activator of lactate production in neuroblastoma cells, but not in normal fibroblasts and neuronal cells. Moreover, the reducing effect of tetrathiomolybdate on cell viability and the level of ATP in the cells with inhibited Akt kinase/glucose uptake was also selective for neuroblastoma cells. Therefore, efficient elimination of neuroblastoma cells requires inhibition of both glucose uptake/Akt kinase and oxidative phosphorylation activities. The use of tetrathiomolybdate as a mitochondrial inhibitor contributes to selectivity of this combined treatment, preferentially targeting neuroblastoma cells.

## Introduction

Neuroblastoma is a solid tumour arising from neural crest cells of the sympathetic nervous system 1. It is a severe form of childhood cancer, by far the most common type of tumour amongst infants responsible for more than 7% of malignancies in children under the age of 15. Survival rate reaching 84% in infants decreases with age 2. Poor survival rates of high‐risk patients demand searching for novel therapeutic strategies 3.

Cancer cells including neuroblastoma display Warburg effect and glucose dependency 4. There are multiple proto‐oncogenes such as Akt, c‐Myc, HIF1alpha, BCR‐ABL, KRAS, BRAF, NRAS, EGFR and ERBB2/HER2 with transforming capacity partially dependent on increased glucose transport and metabolism 5, 6, 7, 8, 9. Inhibition of the oncogene‐stimulated glucose metabolism might provide a base for therapy of the disease 6, 8. Indeed, inhibition of glycolysis by either glycolysis inhibitors or dietary modification had been proposed for neuroblastoma therapy earlier 10, 11, 12.

Akt is a serine/threonine kinase that is often up‐regulated in a variety of cancers. The Akt activity depends on phosphorylations at the catalytic domain (Akt Thr308) and the C‐terminal regulatory domain (Akt Ser473) 13, 14. Phosphorylation of Akt at Thr308 results from activity of serine–threonine phosphoinositide‐dependent kinase 1 (PDK1) 15. Akt Thr308 up‐regulates activity of mTORC1 and p70S6K and enhances cell proteosynthesis 16. Phosphorylation of Akt at Ser473 by mTORC2 17 promotes anti‐apoptotic and cell survival pathways 18 and stimulates translocation of GLUT4 and transport of glucose 19. There are several isoforms of Akt. Akt1 is expressed in various cells and participates in control of their growth and proliferation 20. Akt2 is involved in control of cell metabolism, as Akt2‐null mice develop insulin resistance and diabetic‐like syndromes 21. Akt3 was proposed to be involved in brain development 22. Akt phosphorylates a myriad of proteins participating in protein translation, metabolism, cell survival/anti‐apoptotic signalling and cell cycle progression 18. As Akt is often hyperactivated in various cancers including high‐risk neuroblastomas, this feature was proposed as a novel prognostic indicator of overall survival. Akt kinase inhibitors were identified as promising tools for treatment of malignant diseases 23, 24, 25, 26, 27. One of these inhibitors, Akti‐1/2, suppresses activation of Akt1 and Akt2 isoforms, thus reducing the level of active Akt in cells. Moreover, it also inhibits glucose transport into cells 28, 29.

Targeting the oncogene‐driven signalling pathways is a clinically validated approach for several types of cancers. Nevertheless, frequent relapses and chemotherapy resistances indicate that a fraction of tumour cells survives the shutdown of oncogenic signalling, suggesting that single‐agent chemotherapy is unlikely to have a radical therapeutic impact 30. The oxidative phosphorylation (OXPHOS) activity might be responsible for rescue of cells with down‐regulated oncogene signalling 31. Although general reduction in mitochondrial respiration seems to occur in neuroblastoma cells 32, mitochondrial inhibitors, such as rotenone (Rot) and meta‐iodobenzylguanidine (MIBG), can further reduce production of ATP 33, 34. In addition, neuroblastoma cells with N‐Myc amplification that is highly associated with advanced stage, aggressive growth and poor prognosis of the disease rely on OXPHOS to satisfy most of their energy demands 35, 36. This suggests that neuroblastoma is not an exclusively glycolytic tumour. The effect of sustained OXPHOS on neuroblastoma cell viability under conditions of glycolysis suppression is a controversial issue, and thus, it deserves further investigation.

In this work, we identified OXPHOS as an important source of intracellular ATP and a factor responsible for maintaining viability of neuroblastoma cells with inhibited glucose uptake/Akt kinase activity. The OXPHOS inhibitors, such as rotenone (Rot) and tetrathiomolybdate (TTM), decreased intracellular level of ATP in these cells and synergized with inhibitor of the glucose uptake/Akt kinase in reduction in cell viability. We also identified TTM as a highly specific inhibitor of oxygen consumption and activator of lactate production in neuroblastoma, but not in normal fibroblasts and neuronal progenitors. Consequently, when used in combination with the Akt kinase/glucose uptake inhibitor, TTM down‐regulated viability and intracellular level of ATP preferentially in neuroblastoma cells leaving normal fibroblasts and neuronal progenitors less affected. This observation is promising for design of future strategies to selectively eliminate tumour cells in anti‐neuroblastoma therapies.

## Materials and methods

### Cell culture

SH‐SY5Y (ECACC, 94030304) and SK‐N‐BE(2) (ATCC, CRL‐2271) cells were cultivated in the HEPES‐modified RPMI 1640 medium (Sigma‐Aldrich, Prague, Czech Republic) supplemented with 10% FCS (Sigma‐Aldrich), L‐glutamine (2 mM), penicillin (100 U/ml) and streptomycin (100 μg/ml; Lonza, Verviers, Belgium) in a humidified 5% CO_2_ atmosphere at 37°C. For multicellular spheroids formation, the SK‐N‐BE(2) cells were seeded at density of 10,000 cells/ml on the 24‐well plate and incubated on a rotary shaker (100 r.p.m., Orbital Shaker, NB‐101SRC, N‐BIOTEK, Korea) in a humidified 5% CO_2_ atmosphere at 37°C for 4 days. Human foreskin fibroblasts (ATCC, SCRC 1041) were cultivated in the DMEM high‐glucose medium with sodium pyruvate supplemented with 15% FBS, L‐glutamine (2 mM), penicillin (100 U/ml), streptomycin (100 μg/ml), non‐essential amino acids and 0.01 mM beta‐mercaptoethanol (both from Sigma‐Aldrich).

Neuronal cells were prepared according to Bartova *et al*., 2016 37. Neural stem/progenitors cells (NSCs) were isolated from the embryonic ganglionic eminence (GE) of the forebrain of C57/BL6 mice at 13.5 dpc. The isolated tissue was gently trypsinized, and the cells were dissociated through mechanical trituration. Aliquots of 40 000 cells/ml were seeded in tissue culture dishes in serum‐free DMEM/F12 (1:1) containing 1 × ITS (insulin, transferrin and selenium), N2 and B27 supplements (all purchased from Gibco–Invitrogen, Carlsbad, CA, USA), 100 U/ml penicillin, 0.1 mg/ml streptomycin, 5 ng/ml FGF‐2 and 20 ng/ml EGF (PeproTech, Rocky Hill, NJ, USA). The neurospheres were passaged every 4th day. Differentiation of the NSCs was induced as follows: neurospheres were trypsinized and resulting single cells at density 20,000 cells/ml were seeded to gelatinized tissue culture plastic in DMEM/F12 (1:1) media containing 1 × ITS supplement, 2% FCS and antibiotics. The cultures were maintained in 5% CO_2_ in a humidified atmosphere at 37°C for 4 days. The differentiating neuronal cells were treated as described in individual experiments. For all experiments, neuroblastoma and neuronal cells were seeded at density 20,000 cells/ml and fibroblasts at 10,000 cells/ml.

### Determination of cell proliferation and viability

The monolayer cells and the multicellular spheroids were treated with either ammonium tetrathiomolybdate (TTM; Sigma‐Aldrich) dissolved in deionized water, rotenone (Rot; Sigma‐Aldrich), the Akt1/2 kinase inhibitor (Akti‐1/2; Santa Cruz Biotechnology, Inc., Dallas, TX, USA) dissolved in DMSO or their combinations. As a control, the cells were left untreated. Total number of living cells was determined by the crystal violet staining as described elsewhere 38. Following incubation, the cells floating in the medium were confirmed to be dead by eosin staining. The medium was removed and the cells attached to the bottom of the plate were washed with cold phosphate‐buffered saline (PBS). Adherent cells were fixed and stained in solution containing 0.05% w/v crystal violet, 1% formaldehyde, 1 × PBS and 1% methanol for at least 20 min. at room temperature. Excess of crystal violet was removed by several washes with water. Cell culture plates were dried overnight. The absorbance of the dissolved dye corresponding to the number of living cells was measured in a microplate reader at 570 nm. To determine the colony‐forming efficiency of the cells cultivated in 3D conditions, multicellular spheroids were first disintegrated in the EDTA/trypsin solution. Next, single‐cell suspensions were seeded in the plates and cultivated for 14 days. Colonies were visualized by crystal violet staining and enumerated by light microscopy. Colony‐forming efficiency was expressed as the number of colonies divided by the number of the cells seeded ×100 39.

To evaluate cell survival by flow cytometric quantification of fluorescein diacetate/propidium iodide staining 40, the cells were treated with fluorescein diacetate (0.05 μg/ml) and propidium iodide (1 μg/ml) in the dark for 10 min. at 37°C, then placed on ice and kept ice‐cold during the flow cytometric analysis of red/green fluorescence. Living cells were identified as those with high green/low red fluorescence (FITC positive/PE negative) and dead cells as those with low green/high red fluorescence (FITC negative/PE positive).

### Assessment of mitochondrial membrane potential

The cationic dye JC‐1 forms multimeric aggregates in mitochondria with high membrane potential; these aggregates emit light in the high orange wavelength of 590 nm when excited at 488 nm. In mitochondria with low membrane potential, JC‐1 forms monomers that emit light in the green wavelength (525–530 nm) when excited at 488 nm. The cells treated with Akti‐1/2 were stained with JC‐1 (Biotium, Fremont, CA, USA) according to the manufacturer recommendations and incubated at 37°C in the dark for 40 min. before analysis by flow cytometry.

### Determination of glucose and lactate concentrations in media

SK‐N‐BE(2), SH‐SY5Y, non‐malignant fibroblasts and neuronal cells were treated with either Akti‐1/2, Rot or TTM. As control, the cells were left untreated. Media conditioned with control and treated cells were collected and analysed for glucose and lactate concentrations using commercial kits (Glucose Assay Kit; BioVision, Mountain View, CA, USA, and Lactate Assay Kit; Cayman Chemical Company, Ann Arbor, MI, USA). To determine the glucose consumption/lactate production, basal glucose/lactate levels in cultivation media were assessed and subtracted from the glucose/lactate levels determined in the cell‐conditioned media. The results were normalized according to the number of living cells enumerated using crystal violet staining.

### Determination of intracellular concentration of ATP

The cells were treated with Akti‐1/2, TTM, Rot and their combinations. Following incubation, adherent cells were harvested and intracellular concentration of ATP was determined using ATP Colorimetric/Fluorometric Assay Kit (BioVision) and normalized according to the protein concentration determined by DC protein assay (Bio‐Rad, Hercules, CA, USA).

### Determination of oxygen consumption and media acidification

The cells were treated with TTM and Rot and cultivated in OxoDish^®^ and HydroDish^®^. The SDR SensorDish^®^ Reader (PreSens—Precision Sensing GmbH, Regensburg, Germany) was used to monitor changes of either dissolved oxygen (% air saturation) or pH of the cultivation medium. As a control, the cell‐free medium was used. Oxygen consumption was calculated as the difference between the oxygen level in the medium alone and in medium conditioned by the cells and normalized to the number of living cells determined by eosin staining.

### Gel electrophoresis and immunoblotting

The cells were lysed by boiling in SDS‐loading buffer containing 0.1 M Tris (pH 6.8), 16% v/v glycerol, 3.2% w/v SDS, 10% v/v β‐mercaptoethanol and 0.005% w/v bromophenol blue. Sample loading was normalized according to protein concentration determined by DC protein assay (Bio‐Rad). Cell lysates were subjected to SDS‐PAGE and electroblotted to PVDF membrane. The blots were probed with the pAkt (Ser473)‐, Akt‐, p‐p70S6K (Thr421/Ser424)‐ and pGSK3beta (Ser9)‐specific antibodies (Cell Signaling Technology, Beverly, MA, USA) or the anti‐alpha‐tubulin antibody (Sigma‐Aldrich) according to instructions of manufacturers. The blots were developed using secondary antibodies conjugated to peroxidase (Cell Signaling Technology; Sigma‐Aldrich) and standard ECL using Immobilon Western Chemiluminescent HRP Substrate (Millipore, Billerica, MA, USA).

### Statistics

Values were expressed as means ± S.D. To determine statistical significance, values were compared by a two‐tailed t‐test for unpaired samples. Differences were considered to be statistically significant if *P*‐value < 0.05. All results were repeated in at least three independent experiments.

## Results

### Majority of SK‐N‐BE(2) neuroblastoma cells remain viable when exposed to the Akt kinase/glucose uptake inhibitor

**Figure 1:**
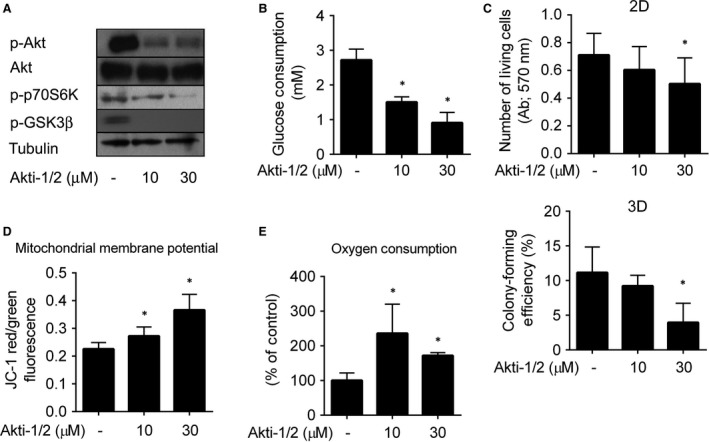
Metabolic adaptation of SK‐N‐BE(2) neuroblastoma cells suppresses cytotoxicity of Akti‐1/2 inhibitor. (**A**) Cells treated with Akti‐1/2 or solvent for 6 hrs were harvested, and extracted proteins were resolved by SDS‐PAGE and analysed by immunoblotting. (**B**) Conditioned media of the cells treated with Akti‐1/2 or solvent for 24 hrs were collected and analysed for glucose concentrations. The columns indicate glucose consumption relative to the number of living cells. (**C**) Viability of the cells treated with Akti‐1/2 or solvent for 24 hrs was determined by crystal violet staining (2D graph). Multicellular spheroids of SK‐N‐BE(2) were formed, treated with Akti‐1/2 or solvent for 24 hrs, and the clonogenic capacity of individual spheroid‐forming cells was determined (3D graph). (**D, E**) Cells were treated with Akti‐1/2 or solvent for 24 hrs, the mitochondrial membrane potential (**D**) and the oxygen consumption (**E**) were assessed as described in Materials and methods. Asterisks indicate significant differences from untreated controls (*P* < 0.05).

The anti‐apoptotic and proliferation promoting effects of the Akt kinase are dependent on uptake and metabolism of glucose 18, 41. Inhibition of Akt and block of the glucose uptake in cancer cells were proposed to be effective strategies for therapy of neuroblastoma 42. We confirmed by SDS‐PAGE and immunoblotting that in SK‐N‐BE(2) cells, the Akt‐1/2 kinase inhibitor effectively suppressed the active form of Akt (pAkt Ser473), the cell growth‐promoting p70S6K as well as the Akt‐regulated pro‐apoptotic GSK3β protein 18, 43, 44 (Fig. 1A). To test the effect of Akti‐1/2 on uptake of glucose, we treated SK‐N‐BE(2) cells with Akti‐1/2 for 24 hrs. We found that in SK‐N‐BE(2) cells, Akti‐1/2 in 10 μM concentration significantly inhibited the glucose uptake by about 50% (Fig. 1B). This effect was even stronger when Akti‐1/2 was used in 30 μM concentration. These results confirm that Akti‐1/2 is an effective inhibitor down‐regulating levels of the pAkt, p‐p70S6K, p‐GSK3β proteins as well as the extent of glucose uptake by SK‐N‐BE(2) cells.

To determine the effect of Akti‐1/2 on cell viability, the cells were treated with Akti‐1/2 in 2D setting for 24 hrs, and the number of living cells was determined by crystal violet staining. To better simulate conditions *in vivo*, we performed cytotoxicity assays also in 3D conditions using spheroids of SK‐N‐BE(2) cells. The spheroids were treated with Akti‐1/2, disaggregated to single‐cell suspension, and clonogenic capabilities of the cells were assessed. In 10 μM concentration, Akti‐1/2 did not reduce number of viable SK‐N‐BE(2) cells or their colony‐forming efficiency significantly (Fig. 1C). However, in 30 μM concentration, Akti‐1/2 significantly decreased number of living cells by 28% and colony‐forming efficiency by 64% of controls (Fig. 1C). To confirm the results obtained by crystal violet staining, the cells treated with Akti‐1/2 in 10 and 30 μM concentrations for 24 hrs in 2D setting were stained with FDA/PI, and frequency of living cells and dead cells was quantified by flow cytometry. Akti‐1/2 did not significantly reduce frequency of living cells in 10 μM concentration. In 30 μM concentration, frequency of living cells dropped by 25% (Fig. 1), thus reaching statistical significance. These results document that significant portion of SK‐N‐BE(2) cells can adapt to the presence of the Akt inhibitor and avoid its cytotoxic effect, presumably due to activation of an alternative survival pathway.

As preservation of mitochondrial function might support viability of cancer cells with inhibited glucose uptake and oncogene signalling 30, we measured the mitochondrial membrane potential and oxygen consumption in the Akti‐1/2‐treated SK‐N‐BE(2) cells. We observed that the Akti‐1/2‐treated cells increased both their mitochondrial membrane potential and oxygen uptake (Fig. 1D andE), suggesting that mitochondrial metabolism was preserved and even elevated in SK‐N‐BE(2) cells with inhibited uptake of glucose and the Akt kinase activity.

### Combined inhibition of Akt and OXPHOS blocks growth of neuroblastoma cells in a synergistic manner

**Figure 2:**
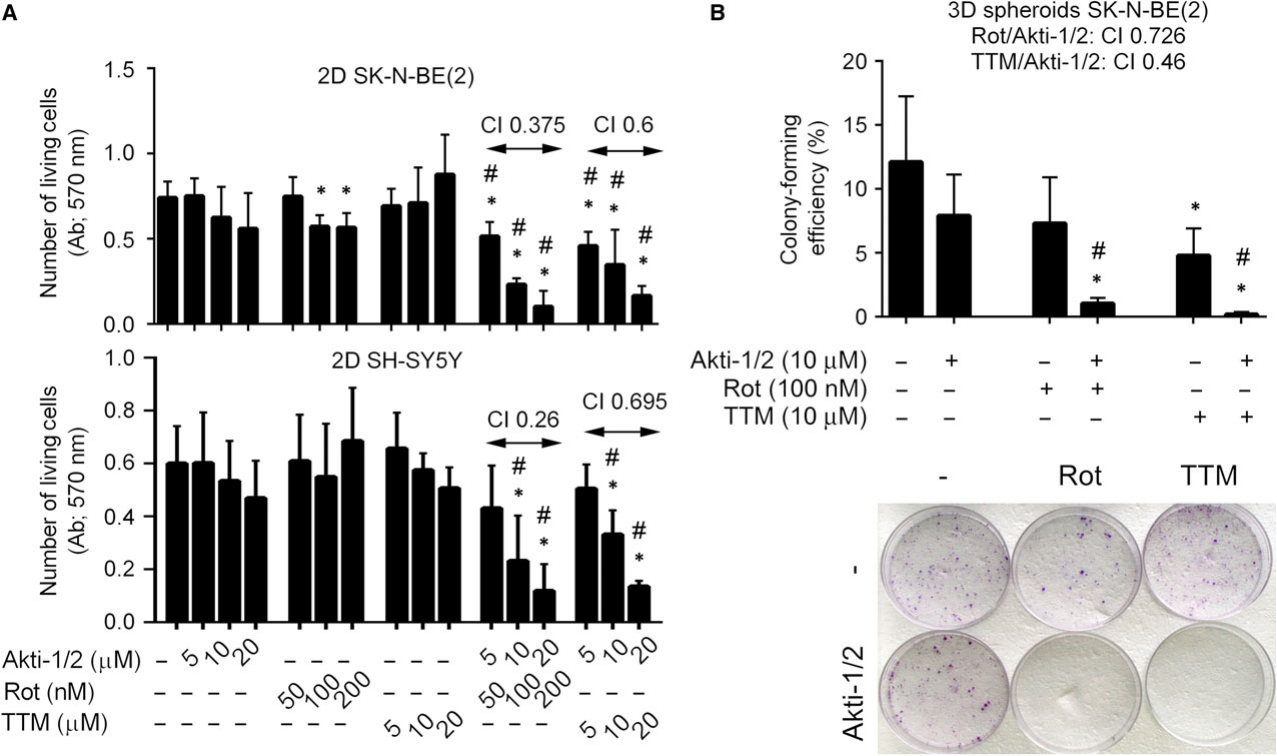
Mitochondrial inhibitors enhance cytotoxicity of Akti‐1/2 to neuroblastoma cells in 2D and 3D conditions. (**A**) SK‐N‐BE(2) and SH‐SY5Y cells were either pretreated with TTM for 24 hrs before Akti‐1/2 was added for the following 24 hrs or treated with Rot/Akti‐1/2 for 24 hrs. Number of living cells was determined by crystal violet staining, and the combination index (CI) was determined. (**B**) The 3D multicellular spheroids of SK‐N‐BE(2) cells were generated and treated as described above (**A**). The clonogenic capacity of individual spheroid‐forming cells and the CI were assessed. The representative samples of colonies growing from the 3D spheroid‐forming cells are shown in photograph (bottom). Asterisks indicate significant differences from untreated controls (*P* < 0.05), # indicates significant differences between samples treated individually and in combination (*P* < 0.05).

To determine whether OXPHOS represents a survival metabolic pathway active in neuroblastoma cells with inhibited kinase Akt‐1/2 and glucose uptake, we addressed the cytotoxic effect of Akti‐1/2 in combination with mitochondrial inhibitors. We used Rot to inhibit the mitochondrial complex I and TTM to inhibit the copper‐dependent mitochondrial respiratory chain complex IV. SK‐N‐BE(2) and SH‐SY5Y cells were treated with Akti‐1/2 (5, 10, 20 μM), Rot (50, 100, 200 nM) and their combination or Akti‐1/2 (5, 10, 20 μM), TTM (5, 10, 20 μM) and their combination. Number of living cells was assessed by crystal violet staining. When used individually, these drugs did not decrease cell viability by more than 23% (Fig. 2A). However, when used in combinations, the effect was more dramatic causing decrease in number of living SK‐N‐BE(2) cells by 68% and 53% by Akti‐1/2/Rot (10 μM/100 nM) and Akti‐1/2/TTM (10 μM/10 μM), respectively. Viability of SH‐SY5Y cells under these conditions dropped by 61% and 55%, respectively (Fig. 2A). Using the Chou–Talalay method 45, the effects of Rot/Akti‐1/2 and TTM/Akti‐1/2 on SK‐N‐BE(2) and SH‐SY5Y cells were found to be synergistic (CI Rot/Akti‐1/2: SK‐N‐BE(2) 0.375, SH‐SY5Y 0.26; CI TTM/Akti‐1/2: SK‐N‐BE (2) 0.6, SH‐SY5Y 0.695) (Fig. 2A).

The enhancement of cytotoxicity resulting from a block of glucose uptake and inhibition of Akt by mitochondrial inhibitors (Rot, TTM) in neuroblastoma cells cultivated in 2D conditions was also verified by FDA/PI staining followed by flow cytometry. We detected a significant down‐regulation of living SK‐N‐BE(2) and SH‐SY5Y cells upon combined treatments with TTM/Akti‐1/2 and Rot/Akti‐1/2 in comparison with controls (Fig. S2). These results show that cytotoxic effect of Akti‐1/2 on neuroblastoma cells can be efficiently stimulated by inhibitors of mitochondrial respiration.

To better simulate conditions *in vivo*, we analysed the synergism between Akti‐1/2 and Rot/TTM as well as Akti‐1/2 and TTM in 3D conditions. Again, we detected synergistic decrease in clonogenic capabilities of the spheroid‐forming cells treated with TTM/Akti‐1/2 or Rot/Akti‐1/2 (CI Rot/Akti‐1/2: 0.726; TTM/Akti‐1/2: 0.46; Fig. 2B).

**Figure 3:**
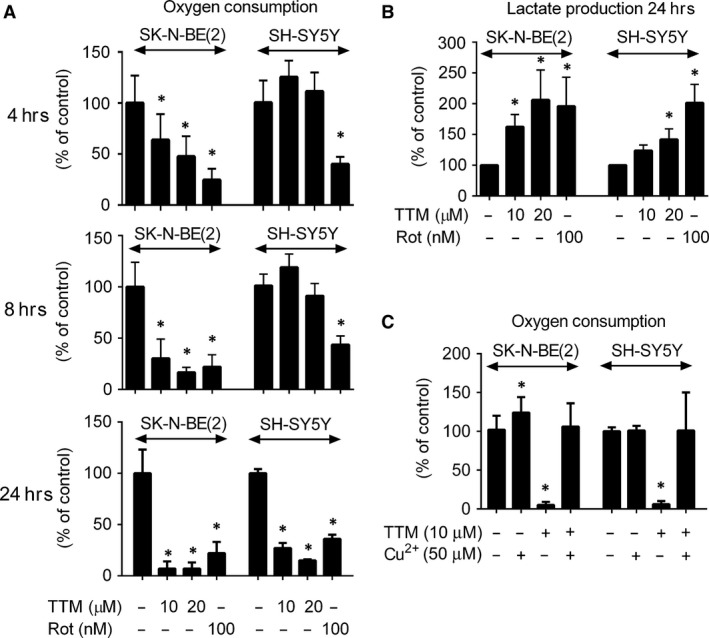
TTM decreases oxygen consumption and increases lactate production in SK‐N‐BE(2) and SH‐SY5Y cells in a copper‐dependent manner. SK‐N‐BE(2) and SH‐SY5Y cells were treated with TTM and Rot for indicated time. (**A, B**) Oxygen consumption (**A**) and lactate production (**B**) by these cells were assessed as described in Materials and methods. (**C**) The cells were similarly treated with TTM (10 μM) and/or Cu^2+^ (50 μM) for 24 hrs, and oxygen consumption was determined. Asterisks indicate significant differences from untreated controls (*P* < 0.05).

Next, we determined the extent of inhibition of oxygen consumption induced in SK‐N‐BE(2) and SH‐SY5Y cells by inhibitors of mitochondrial respiration in concentrations effectively enhancing cytotoxicity of Akti‐1/2. Kinetics of TTM‐induced inhibition of oxygen consumption in SK‐N‐BE(2) cells was faster than in SH‐SY5Y cells, but TTM effectively inhibited oxygen consumption in both cell types within 24 hrs (Fig. 3A). The inhibitory effect of Rot was strong and significant in both cell lines and all time intervals tested (Fig. 3A).

As inhibition of mitochondrial metabolism should increase production of lactate 46, the level of lactate in SK‐N‐BE(2)‐ and SH‐SY5Y‐conditioned media was determined. Indeed, both cell types treated with TTM and Rot increased production of lactate to cultivation media (Fig. 3B). The effect of TTM was repeatedly more dramatic in SK‐N‐BE(2) than in SH‐SY5Y cells (Fig. 3B), suggesting that TTM was a more efficient inhibitor of mitochondrial metabolism in SK‐N‐BE(2) than in SH‐SY5Y cells.

TTM is a well‐established chelator of copper. To verify that SK‐N‐BE(2) cells are more sensitive to perturbations of the copper concentration than SH‐SY5Y cells, we compared oxygen production of these cells upon treatment with Cu^2+^ (50 μM) and TTM (10 μM) for 24 hrs. We found that addition of Cu^2+^ stimulated uptake of oxygen by SK‐N‐BE(2), but not by SH‐SY5Y cells (Fig. 3C). Moreover, supplementation with Cu^2+^ suppressed the effect of TTM in both cell lines (Fig. 3C). These results document that oxygen consumption by SK‐N‐BE(2) cells is more sensitive to fluctuation of copper than by SH‐SY5Y cells.

### Inhibitors of mitochondrial respiration down‐regulate ATP and pAkt in neuroblastoma cells treated with Akti‐1/2

**Figure 4:**
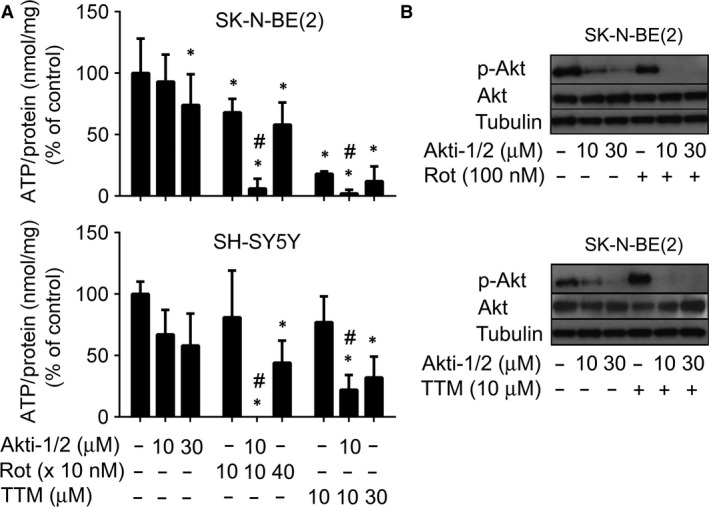
Mitochondrial inhibitors enhance suppressive effects of Akti‐1/2 on ATP production and the pAkt protein formation in neuroblastoma cells. (**A**) SK‐N‐BE(2) and SH‐SY5Y cells were pretreated with TTM for 24 hrs before Akti‐1/2 was added for the following 6 hrs. The same cells were also treated with Rot and Akti‐1/2 for 6 hrs. Intracellular level of ATP in harvested cells was determined, normalized according to protein concentration and expressed as a percentage of untreated controls. Asterisks indicate significant differences from untreated controls (*P* < 0.05); # indicates significant differences between samples treated individually and in combination (*P* < 0.05). (**B**) SK‐N‐BE(2) cells were treated with TTM (10 μM), Rot (100 nM) and Akti‐1/2 as described in (**A**). Samples containing the same amounts of proteins extracted from harvested cells were resolved by SDS‐PAGE and analysed by immunoblotting.

The impact of Akt/OXPHOS inhibitors on cellular metabolism should be reflected in perturbation of intracellular level of ATP. Therefore, we followed the effect of Akti‐1/2, Rot and TTM on the level of ATP in neuroblastoma cells. We found that simultaneous treatment of both cell types with Rot/Akti‐1/2 or TTM/Akti‐1/2 decreased the level of ATP more effectively than these drugs used individually (Fig. 4A). Inhibition of mitochondrial metabolism or decrease in intracellular ATP might affect the level of the active Akt kinase, especially the form phosphorylated at Ser473 47, 48. However, the level of pAkt(Ser473) in SK‐N‐BE(2) cells was not affected by Rot and increased by TTM as determined by immunoblotting (Fig. 4B). In the presence of Akti‐1/2, the pAkt protein completely disappeared from both Rot‐ and TTM‐treated cells (Fig. 4B). These results suggest that inhibition of glucose uptake, Akt kinase activity and OXPHOS is needed to effectively eliminate production of intracellular ATP and pAkt(Ser473) in neuroblastoma cells.

### The effect of TTM on cellular metabolism is specific for neuroblastoma, but not for normal neuronal cells and fibroblasts

**Figure 5:**
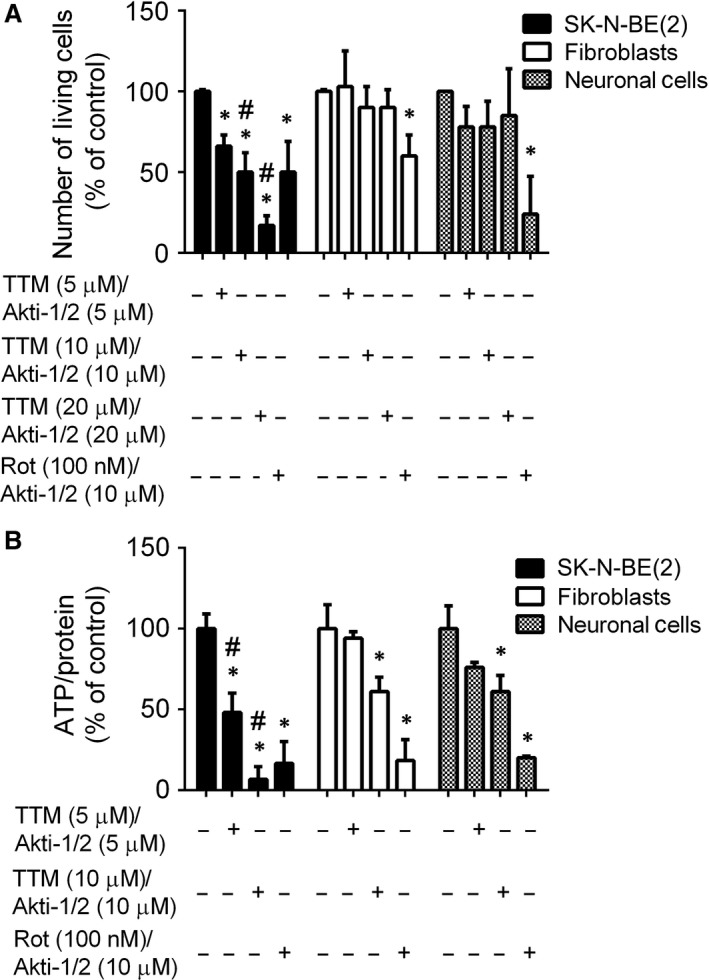
TTM/Akti‐1/2 exhibits stronger cytotoxic and ATP‐depleting activities to neuroblastoma SK‐N‐BE(2) cells than to normal fibroblasts and neuronal precursors. (**A**) SK‐N‐BE(2) cells, normal fibroblasts and neuronal cells were treated as described above (Fig. 2A). Number of living cells was determined by crystal violet staining and (**B**) the intracellular level of ATP was determined as described in the legend of Figure 4A. Data are presented as a percentage of the untreated controls. Asterisks indicate significant differences from untreated controls (*P* < 0.05); # indicates significant differences between similarly treated cancer and normal cells.

**Figure 6:**
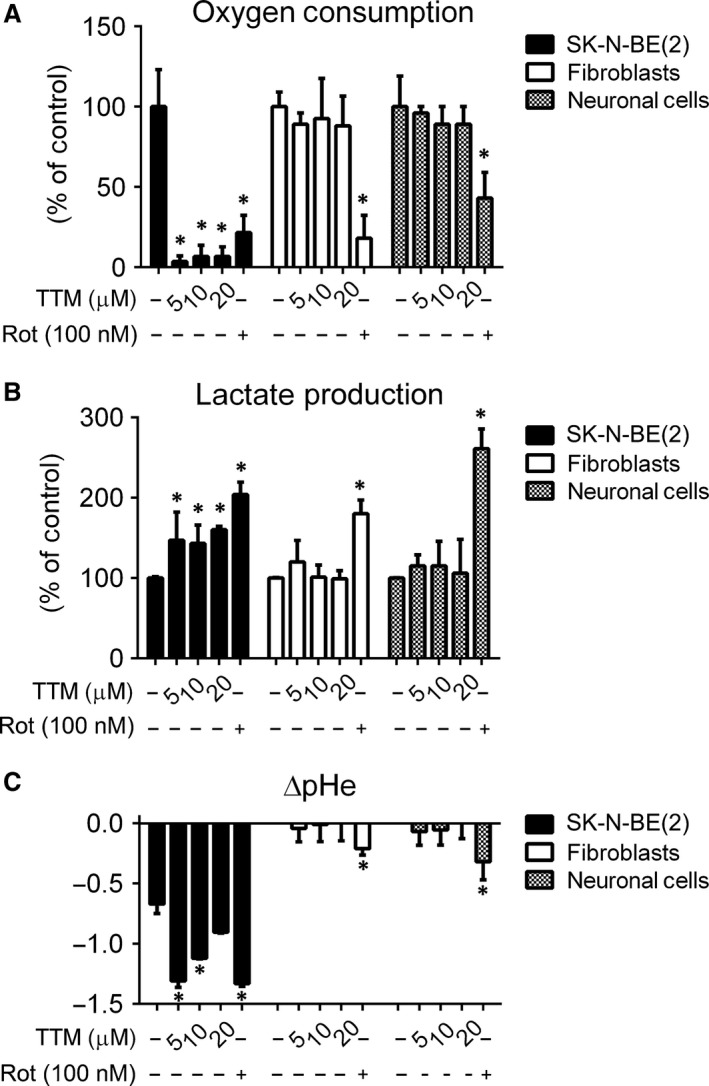
TTM inhibits oxygen consumption and increases extracellular acidification and lactate production in neuroblastoma SK‐N‐BE(2) cells, but not in normal fibroblasts and neuronal precursors. SK‐N‐BE(2) cells, normal fibroblasts and neuronal cells were treated with either TTM or Rot at indicated concentrations for 24 or 48 hrs, respectively. (**A**) The oxygen consumption and (**B**) production of extracellular lactate by cells treated for 24 hrs and (**C**) pHe in cultivation media after 48 hrs of cultivation were determined as described in Materials and methods. Asterisks indicate significant differences from untreated controls (*P* < 0.05).

The need to inhibit multiple metabolic pathways in tumour cells raises the problem of how to avoid toxicity to normal healthy cells. We showed that combined treatment of neuroblastoma cells with both TTM/Akti‐1/2 and Rot/Akti‐1/2 was more cytotoxic than the agents alone. Next, we wished to compare cytotoxicity of these drugs to neuroblastoma and normal cells. Human fibroblasts and neuronal cells were treated with Rot/Akti‐1/2 and TTM/Akti‐1/2 in the concentrations that were shown to effectively reduce viability of neuroblastoma cells in previous experiments. We verified that Rot (100 nM), TTM (5, 10, 20 μM) and Akti‐1/2 (5, 10, 20 μM) were not cytotoxic to normal fibroblasts and neuronal cells when used individually (not shown). However, combined treatment of TTM/Akti‐1/2 was significantly more cytotoxic to SK‐N‐BE(2) cells than to fibroblasts and neuronal cells (Fig. 5A). In contrast, Rot/Akti‐1/2 was cytotoxic to all tested cell types (Fig. 5A). Different effects of TTM/Akti‐1/2 and Rot/Akti‐1/2 on cell viability correlated with their effects on intracellular level of ATP. TTM/Akti‐1/2 (10 μM/10 μM) reduced intracellular ATP by about 40% in normal cells, but about 90% in neuroblastoma SK‐N‐BE(2) cells (Fig. 5B). The effect of Rot/Akti‐1/2 was less specific, decreasing the level of ATP by about 80% in both normal and neuroblastoma cells (Fig. 5B). These results suggest that combination of TTM and Akti‐1/2 can specifically target cancer cells leaving the normal cells less affected. TTM alone can specifically target neuroblastoma cells as demonstrated by its strong inhibitory effect on oxygen consumption by SK‐N‐BE(2) cells and lack of this effect on normal fibroblasts and neuronal cells (Fig. 6A). In contrast, treatment with Rot was less selective, inhibiting oxygen consumption by both normal and cancer cells (Fig. 6A). Inhibition of mitochondrial function is often associated with changes in extracellular pH (pHe) and/or increased production of lactate 49. Therefore, we determined these parameters in SK‐N‐BE(2), normal fibroblasts and neuronal cells. TTM treatment significantly increased extracellular level of lactate and decreased pHe in SK‐N‐BE(2), but not in normal fibroblasts and neuronal cells. In contrast, Rot treatment increased extracellular level of lactate and decreased pHe in all tested cell types (Fig. 6B and C). The effects of TTM and Rot on oxygen consumption by SK‐N‐BE(2) cells, fibroblasts and neuronal cells and their pHe in real time are illustrated in Figure S3 These results imply that TTM is a selective inhibitor of mitochondrial respiration in neuroblastoma cells. In addition, in combination with Akti‐1/2, it can specifically eliminate neuroblastoma cells leaving normal healthy cells significantly less affected.

## Discussion

This study shows that inhibition of glucose uptake and Akt reduces number of living neuroblastoma SK‐N‐BE(2) and SH‐SY5Y cells only partially (20–30%). Therefore, we aimed to identify and target alternative survival promoting pathways to increase cytotoxic effect of glucose uptake/Akt kinase inhibition in these cells. As described earlier, inhibition of the oncogene‐driven signalling pathways such as KRAS, MEK1/PI3K‐mTOR, BRAF, hexokinase 2 and BCR‐ABL in various cancers can restore OXPHOS and select for tumour cells responsible for relapse of the disease 31, 50, 51, 52. The Akt kinase can activate both glycolysis and mitochondrial respiratory capacity in cells 8, 53. However, down‐regulation of Akt in neuroblastoma SK‐N‐BE(2) cells was not associated with reduction in the OXPHOS activity. In contrast, increased mitochondrial potential and oxygen consumption in these cells suggested elevation of mitochondrial activity. The importance of OXPHOS for generation of ATP in neuroblastoma remains a controversial issue. It may be cell type specific or dependent on the type of metabolic perturbation. For example, neuroblastoma xenografts derived from SK‐N‐BE(2) and SH‐SY5Y cells exhibit low mitochondrial OXPHOS activity and inability to increase the mitochondrial OXPHOS activity in response to calorie restriction/ketogenic diet 11. In contrast, restriction of glycolysis flux increases mitochondrial respiration in SH‐SY5Y cells 54. Similarly, neuroblastoma N2a cells produce a considerable amount of ATP by OXPHOS 55. It has been proposed that a history of the carcinogenic process in concert with tumour‐specific microenvironment determines the final metabolic phenotype of selected cancer cells 49.

Even if neuroblastoma cells are able to use/reactivate OXPHOS due to inhibition of glycolysis or low glucose environment, to our knowledge, the impact of OXPHOS on their viability has not been systematically addressed yet. It is generally accepted that reactivation of OXPHOS in cancer cells can either sustain their viability or induce apoptosis 31, 56, 57, 58. Thus, we addressed the question whether OXPHOS is important for survival of neuroblastoma cells lacking the Akt kinase activity with suppressed glucose uptake. Rot and TTM, OXPHOS inhibitors 34, 59, 60, increased cytotoxicity of Akti‐1/2 on neuroblastoma cells in a synergistic manner. As monolayer cultures might not adequately represent the three‐dimensional (3D) physiological behaviour of neuroblastoma *in vivo* and proteins involved in glycolysis, cell stress, antioxidant defence, cell structure and signal transduction are differentially expressed in neuroblastoma monolayers and spheroids 61, we tested cytotoxicity of TTM/Akti‐1/2 and Rot/Akti‐1/2 on neuroblastoma multicellular tumour spheroids. Again, even in the 3D model, we confirmed synergistic cytotoxicity of glucose uptake/Akt kinase inhibition and mitochondrial inhibitors (Rot, TTM). These results suggested that it is the OXPHOS activity that interferes with cytotoxicity of the Akt kinase/glucose uptake inhibitor on neuroblastoma SK‐N‐BE(2) and SH‐SY5Y cells.

The level of intracellular ATP is an important marker of cellular metabolism. We detected only a minor reduction in ATP level in the neuroblastoma cells treated with Akti‐1/2. This result was unexpected as neuroblastoma SK‐N‐BE(2) and SH‐SY5Y cells were reported to rely mostly on glycolysis for ATP production 11. Nevertheless, as actual intracellular level of ATP results from the balance between the ATP production and consumption 62, inhibition of the ATP‐dependent synthesis of macromolecules might lower consumption and preserve the level of ATP in neuroblastoma cells treated with Akti‐1/2. Indeed, we observed down‐regulation of the p‐p70S6K protein in the Akti‐1/2‐treated SK‐N‐BE(2) cells suggesting suppression of proteosynthesis 44. This indicates that ceasing the macromolecular synthesis might be a rapid cellular adaption to preserve ATP in cells with inhibited glucose uptake/Akt kinase activity 62. Importantly, even in cells with partially blocked proteosynthesis, sustained activity of OXPHOS is important for preserving cellular viability 62. Therefore, we determined the effect of OXPHOS inhibitors on ATP level in cells lacking Akt activity. We detected rapid decrease in the intracellular ATP (to less than 10% of control) in the cells treated with Rot (100 nM)/Akti‐1/2 (10 μM). Similar results were obtained also for TTM/Akti‐1/2. Thus, retaining OXPHOS activity is an important prerequisite for sustaining sufficient level of ATP in SK‐N‐BE(2) and SH‐SY5Y cells with inhibited glucose uptake/Akt kinase.

The important aspect of cancer therapy is its safety to normal cells. In our experiments, TTM/Akti‐1/2 was significantly more cytotoxic to SK‐N‐BE(2) cells than to non‐malignant fibroblasts and neuronal cells. In contrast, the effect of Rot/Akti‐1/2 was less selective. In the absence of Akti‐1/2, TTM also specifically targeted the cancer cells, inhibiting oxygen consumption and activating lactate production in neuroblastoma but not in normal cells, whilst the effect of Rot was not selective for cancer cells. The explanation of the TTM specificity can be based on metabolism of Cu^2+^ 60, 63. We observed that inhibition of oxygen consumption by TTM can be completely suppressed by external copper supplementation. The inhibitory effects of TTM on mitochondria of neuroblastoma cells presumably result from down‐regulation of the copper‐dependent cytochrome c oxidase (COX) representing the terminal complex of the electron transfer chain and/or the copper‐transporting metal chelators responsible for delivery of Cu^2+^ to COX 38, 59, 63, 64. Although glycolysis is the canonical pathway for production of ATP in cancer cells, Krebs cycle and OXPHOS are important to satisfy their elevated needs for ATP as well, especially in low glucose conditions of tumour microenvironment 30, 38, 65. Therefore, the enhanced need for Cu^2+^ supply to feed mitochondrial respiration and anabolic metabolism of rapidly growing cancer cells may explain TTM selectivity to neuroblastoma cells. Copper was reported to be a limiting factor for cancer growth and OXPHOS, and even the well‐known Warburg effect occurring in tumours was suggested to reflect insufficient copper bioavailability in the tumour microenvironment 64. Numerous studies have reported that both serum ceruloplasmin and copper levels are elevated in a variety of malignancies, including solid tumours and haematological malignancies. Increased level of copper was also shown to directly correlate with cancer progression 66. Inhibition of copper transport proteins AtoxI and CCS by small molecular inhibitor DC_AC50 can reduce growth of lung, leukaemia, breast and head/neck cancer cells without affecting normal tissues in mice 67. Therefore, increased demand of cancer cells for copper supplementation might explain the enhanced effectivity of copper‐depleting TTM on neuroblastoma cells and its safety to normal fibroblasts and neuronal progenitors. The higher efficiency of TTM on SK‐N‐BE(2) than on SH‐SY5Y cells might result from different extent of the N‐Myc amplification and p53 expression in these cell lines. The p53 wt protein that is produced by SH‐SY5Y but not SK‐N‐BE(2) cells contributes to maintenance of the mitochondrial OXPHOS machinery by induction of expression of Sco2. This gene codes for a protein chaperone and a major transporter of copper to COX 68, 69. Loss of this copper transporter in SK‐N‐BE(2) cells due to the absence of active p53 might contribute to their elevated vulnerability to copper‐depleting agents, such as TTM. Apart from the loss of p53 wt, SK‐N‐BE(2) cells display amplification of the N‐Myc oncogene. The N‐Myc‐expressing tumours satisfy a substantial part of their energy demands using OXPHOS with glutamine as a substrate 36. Therefore, cancer cells overexpressing N‐Myc might exhibit elevated demand for copper to supplement the copper‐containing OXPHOS enzymes. In our experiments, Cu^2+^ increased consumption of oxygen in SK‐N‐BE(2), but not in SH‐SY5Y cells. This observation along with enhanced vulnerability of SK‐N‐BE(2) to copper‐depleting TTM suggests enhanced demand of SK‐N‐BE(2) cells for bioavailable copper. Therefore, oxygen consumption‐inhibiting and lactate production‐inducing effects of TTM are stronger in neuroblastoma cells, especially those lacking p53 wt and bearing amplification of N‐Myc than in normal fibroblasts and neuronal cells. To verify this hypothesis, a larger collection of neuroblastoma cell lines with different N‐Myc/p53 expression status has to be investigated.

In conclusion, we showed that OXPHOS is responsible for maintaining intracellular level of ATP and proliferative potential of neuroblastoma cells with inhibited Akt kinase and uptake of glucose. The OXPHOS inhibitors increase cytotoxicity of the Akt kinase inhibitor to these cells in a synergistic manner. Combination of the OXPHOS inhibitor TTM and the glucose uptake/Akt kinase inhibitor Akti‐1/2 specifically inhibits intracellular ATP and viability of neuroblastoma cells whilst leaving normal fibroblasts and neuronal progenitors less affected. This selectivity of TTM can result from the fact that it acts as a selective inhibitor of oxygen consumption and inducer of lactate production in neuroblastoma SK‐N‐BE(2) and SH‐SY5Y cells, but not in normal fibroblasts and neuronal progenitors.

## Conflict of interest statement

Authors declare no conflict of interest.

## Author’s contributions

J. Navrátilová was involved in concept and design; M. Karasová, J. Navrátilová, M. Kohutková Lánová and J. Pacherník were involved in development of methodology; M. Karasová, L. Jiráková, Z. Budková and J. Navrátilová were involved in data acquisition; J. Navrátilová, J. Pacherník, P. Beneš and J. Šmarda were involved in analysis and interpretation of data; J. Navrátilová, J. Pacherník, P. Beneš and J. Šmarda wrote and revised the manuscript; P. Beneš and J. Šmarda were involved in study supervision.

## Supporting information


**Figure S1** Verification of the effect of Akti‐1/2 on viability of neuroblastoma cells by FDA/PI staining.
**Figure S2** Verification of synergistic effect of Akti‐1/2 and mitochondrial inhibitors on viability of neuroblastoma cells by FDA/PI staining.
**Figure S3** Variability of extracellular oxygen and pHe in media conditioned by SK‐N‐BE(2) cells, non‐malignant fibroblasts and neuronal cells.
