# Supplementary material for: Selective elimination of neuroblastoma cells by synergistic effect of Akt kinase inhibitor and tetrathiomolybdate
Source: J Cell Mol Med. 2017 Feb 28;21(9):1859–69. doi: 10.1111/jcmm.13106 (PMC5571524; doi:10.1111/jcmm.13106)
Supplement: Supplementary file 1 — Figure S1 Verification of the effect of Akti‐1/2 on viability of neuroblastoma cells by FDA/PI staining. Figure S2 Verification of synergistic effect of Akti‐1/2 and mitochondrial inhibitors on viability of neuroblastoma cells by FDA/PI staining. Figure S3 Variability of extracellular oxygen and pHe in media conditioned by SK‐N‐BE(2) cells, non‐malignant fibroblasts and neuronal cells. [file JCMM-21-1859-s001.doc]

**Supporting information**


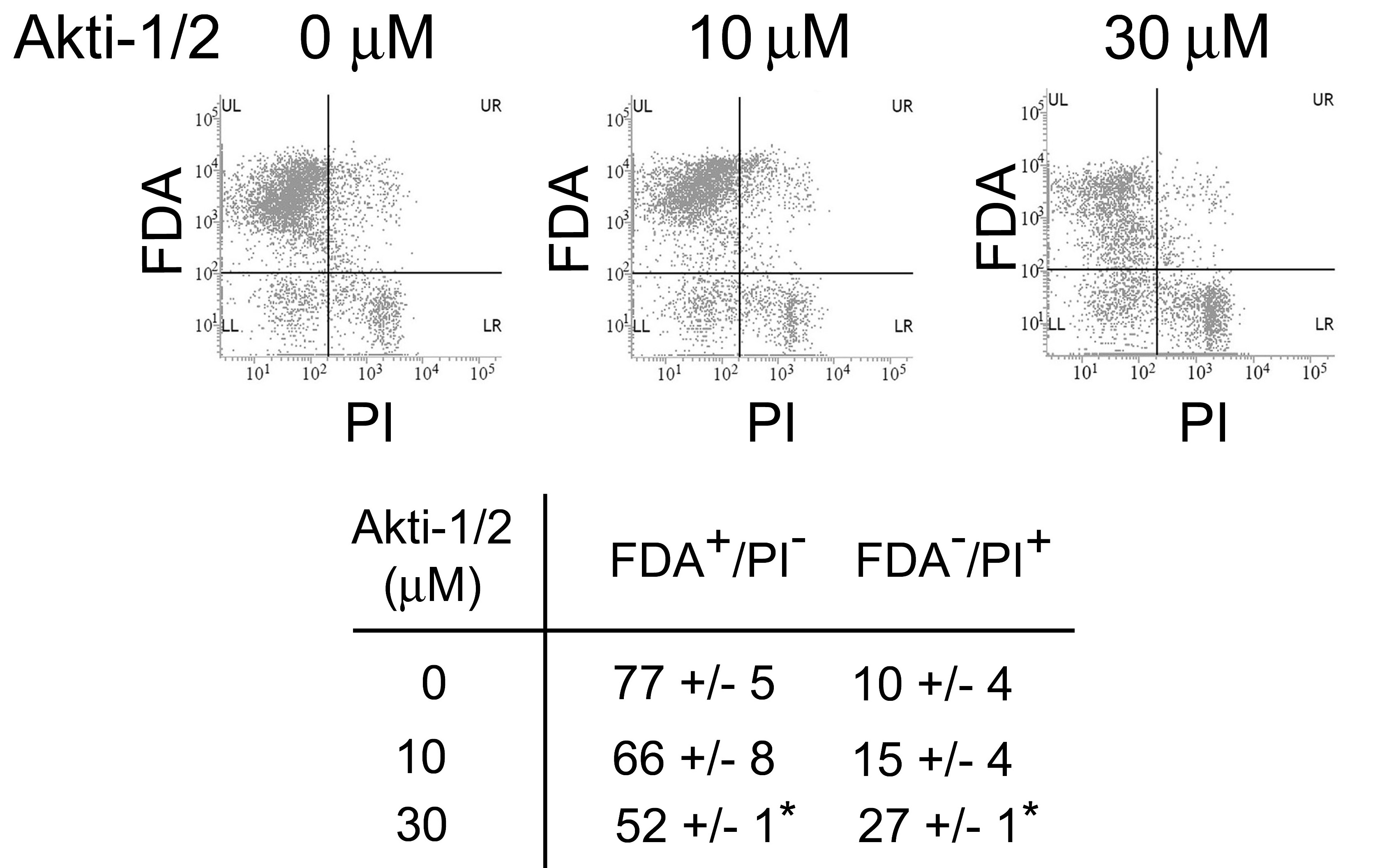


Fig. S1 **Verification of the effect of Akti-1/2 on viability of neuroblastoma cells by FDA/PI staining.** SK-N-BE(2) cells were treated with Akti-1/2 for 24 h. Cells were harvested, stained with FDA/PI and analyzed by flow cytometry. The average frequency of cells (%) exhibiting FDA positivity/PI negativity and FDA negativity/PI positivity +/- standard deviations from three independent experiments are shown. Asterisks indicate significant differences from untreated controls (P < 0.05).


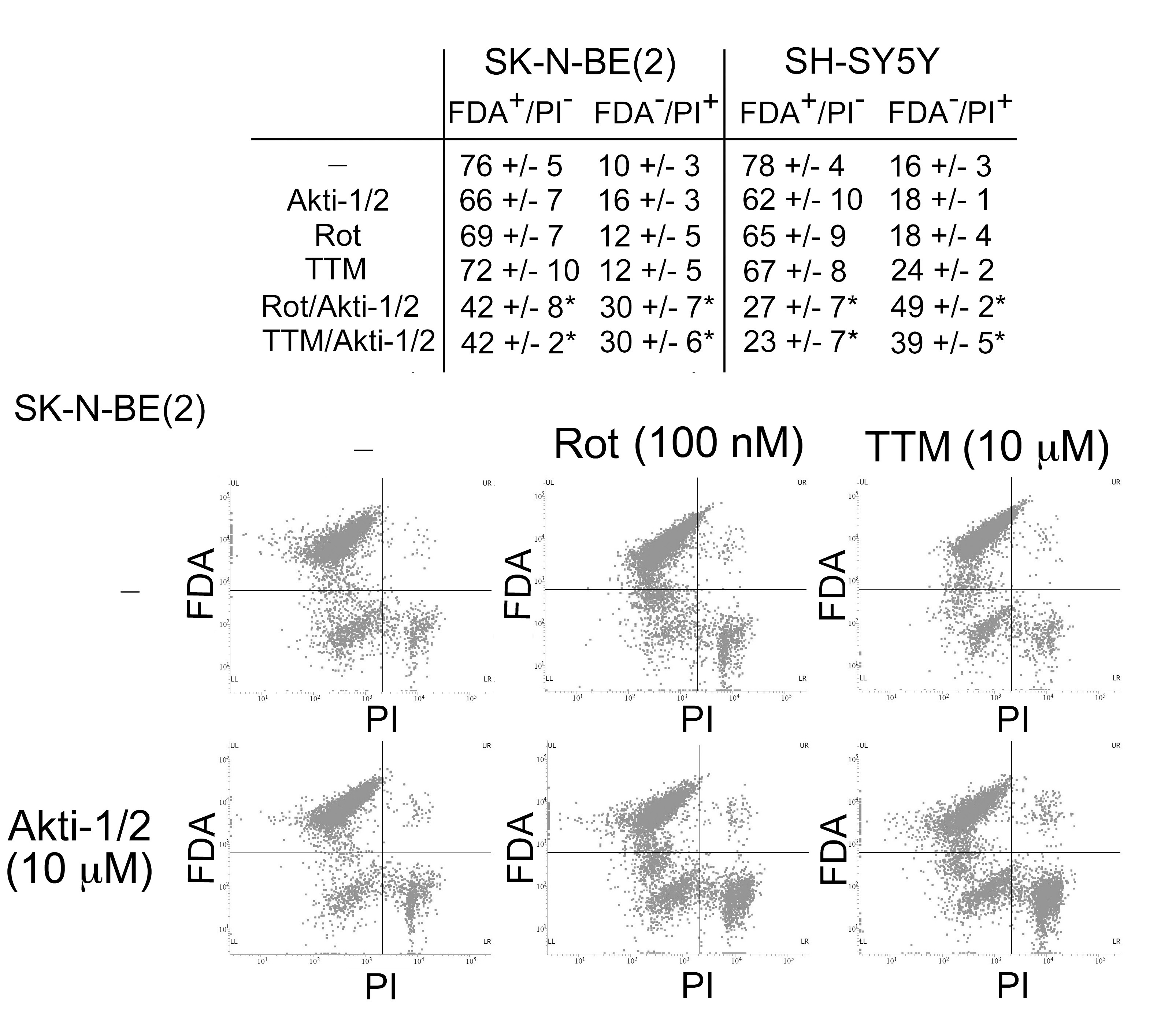


Fig. S2 **Verification of synergistic effect of Akti-1/2 and mitochondrial inhibitors on viability of neuroblastoma cells by FDA/PI staining.** SK-N-BE(2) and SH-SY5Y cells were either pretreated with TTM for 24 h before Akti-1/2 was added for next 24 h or treated with Rot/Akti-1/2 for 24 h. Cells were harvested, stained with FDA/PI and analyzed by flow cytometry. The average frequency of cells (%) exhibiting FDA positivity/PI negativity and FDA negativity/PI positivity +/- standard deviations from three independent experiments are shown. Asterisks indicate significant differences from controls (P < 0.05).


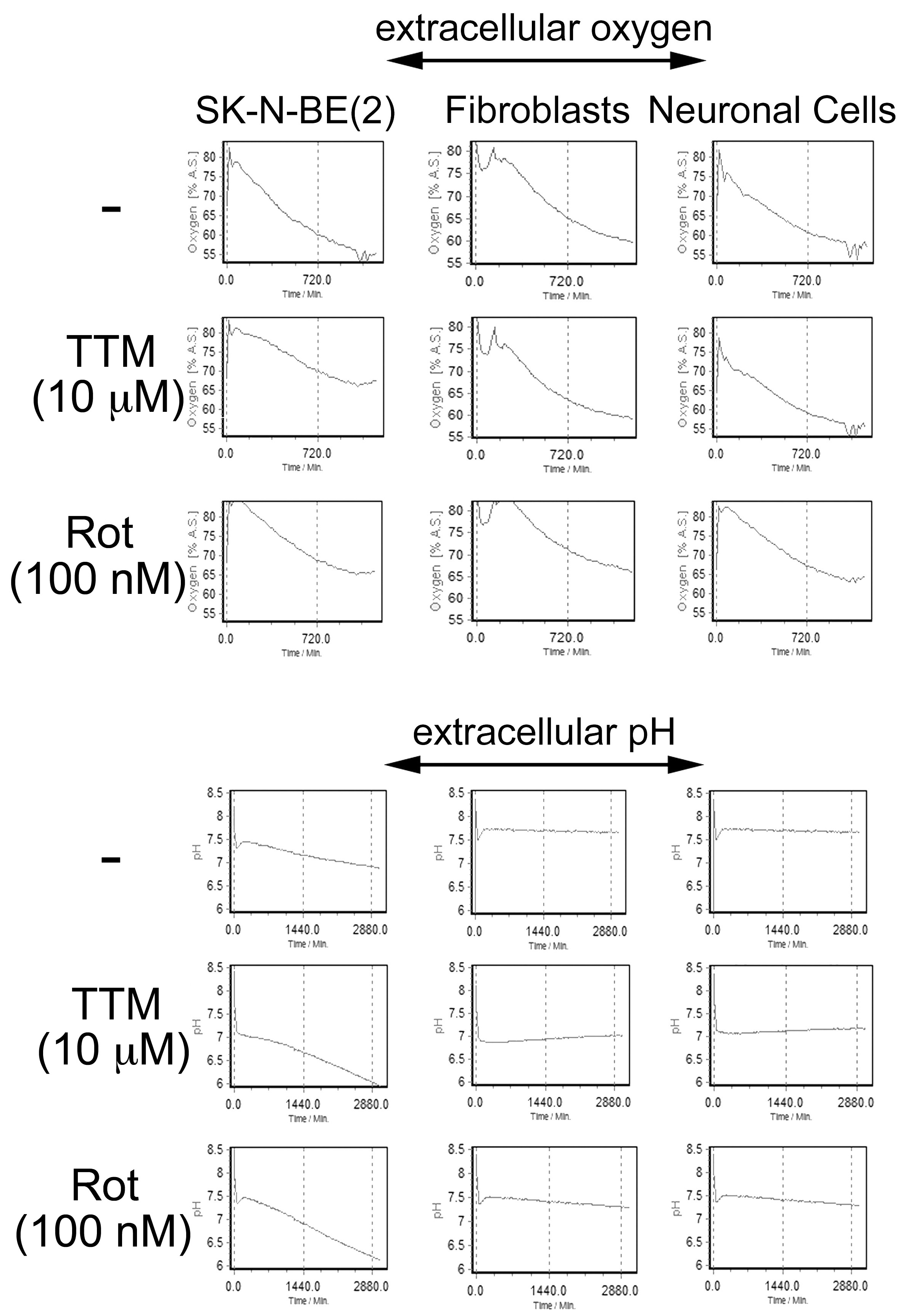


Fig. S3 **Variability of extracellular oxygen and pH** **in media conditioned by SK-N-BE(2) cells, non-malignant fibroblasts and neuronal cells**. SK-N-BE(2) cells, normal fibroblasts and neuronal cells were treated with either TTM and Rot for 24 h (oxygen determination) or 48 h (pH determination). The level of oxygen and pH in the cell-conditioned media were monitored by SDR SensorDish Reader. Graphs show representative results.
